# Assessment of Intravenous Antibiotics to Peroral Antibiotics Conversion Practice and Its Associated Factor at University of Gondar Comprehensive Specialized Hospital: Prospective Observational Study

**DOI:** 10.1155/2022/8395424

**Published:** 2022-10-12

**Authors:** Getachew Yitayew Tarekegn, Samuel Berihun Dagnew, Samuel Agegnew Wondm, Bekalu Kebede, Emneteab Mesfin Ayele

**Affiliations:** ^1^Clinical Pharmacy Unit, Pharmacy Department, Health Science College, DebreTabor University, DebreTabor, Ethiopia; ^2^Clinical Pharmacy Unit, Pharmacy Department, Health Science College, Debre Markos University, Debre Markos, Ethiopia; ^3^Clinical Pharmacy Department, School of Pharmacy, College of Medicine and Health Science, University of Gondar, Gondar, Ethiopia

## Abstract

**Background:**

Improper utilization of antibiotics harms the patient, the public, and the economy. The overuse of injections is one of the key factors in the irrational use of medicines. However, little is known about intravenous (IV) to peroral (PO) conversion practice in the Ethiopian healthcare setting, specifically in the Northwest part of Ethiopia.

**Objective:**

To assess antibiotics IV to PO conversion practice and its associated factors at the internal medicine ward of the University of Gondar Comprehensive and Specialized Hospital (UOGCSH).

**Method:**

A prospective observational study was conducted on 324 study participants who were admitted to the University of Gondar Specialized Hospital from October 3 to November 14, 2021. A systematic random sampling technique was employed to select the study participants. Stata version 14.2 was used for the analysis. Descriptive statistics result was presented using mean and standard deviation. Logistic regression analysis was done to determine the association between independent variables and dependent variables. The association between independent variables and dependent variables was tested at 95% CI and *P* value≤ 0.05 was considered statistical significance.

**Result:**

A total of 324 study participants were included in the study, and the mean age of the patients was 41.4 ± 18.6. Of the 324 study participants, 63.3% were male. The most frequently prescribed antibiotics used for empiric treatment were ceftriaxone (45.4%), followed by metronidazole (33.2%), and cloxacillin (11.4%). A total of 34.5.57% of patients who took antibiotics were converted to PO antibiotics. The most frequently converted type of conversion practice was sequential (23.1%), followed by the switch type of conversion (7.4%). Tachypnea, unavailability of medication, higher temperature, hospital stay greater than 10.78-days, and the presence of comorbidity were predictors of IV medications not being converted to PO medications.

**Conclusion:**

Intravenous to peroral conversion practice was infrequent. The most frequently applied conversion practice was sequential type conversion practice, followed by switch type of conversion practice. IV to PO conversion practice was significantly associated with tachypnea, unavailability of medication, higher temperature, hospital stay greater than 10.78-days, and comorbidity. Awareness of IV to PO conversion practice and short-term training for healthcare teams is vital for better antibiotic conversion practice.

## 1. Introduction

Antibiotic therapy has become vital for the effective management of infectious diseases [1]. A prevalence survey study done in the United States showed that 49.9% of hospitalized patients received at least one antimicrobial drug at the time of the survey [2, 3]. A study in China found that 50.3% of antimicrobials are used in hospitals [4]. The European surveillance study showed that the incidence of antimicrobial use in European countries was 29% [5]. The prevalence of antibiotic use in Egyptians was 59% in 2011 [6].

The ideal route of administration for any medication is to attain the ideal serum concentration without producing any adverse effects [7]. Hence, the safest and most convenient way of medication administration is achieved by the oral route. In addition to this, administration of PO medication achieves tissue and blood concentration to the same extent as that of intravenous medication [8–10]. The availability of oral formulations in the market makes them easier to administer, safe, and achieve the desired therapeutic concentrations [7, 9].

The route of administration of antibiotics, either PO or IV, depends on the patient's condition and site of infection. The PO route of administration has many advantages over the IV route. Among these, it is the most convenient, cheapest available route, easy to use, safe, easy to prepare, and less expensive than the IV route [9, 11]. Appropriate use of antibiotic agents is very important for patient safety and public health [12–16]. One way of optimizing antibiotic use is to switch patients earlier from intravenous to oral therapy as soon as they are clinically stable. This can reduce the length of hospitalization and lower associated costs [17]. On the contrary, injectable antibiotics have so many complications, which include infiltration with an incidence of 31.5% and phlebitis of 29.8% [18]. Despite this disadvantage of using injectable antibiotics for a long period, the literature showed that a high number of antibiotics were not converted into counter-oral routes [19]. To the best of the investigator's knowledge, the prevalence and factors of IV to PO conversion practice were not adequately investigated in resource-limited settings, specifically at the University of Gondar Comprehensive Specialized Hospital. Therefore, the study aimed to investigate the prevalence of IV to PO conversion practice and associated factors.

## 2. Methods

### 2.1. Study Area and Period

An institutional-based prospective observational study was conducted from October 3 to November 14, 2021, in the medical ward of UOGCSH. The hospital is located in Gondar town, North Gondar administrative zone, Amhara regional state. This is located 750 km away from of Addis Ababa, the capital city of Ethiopia. The University of Gondar Hospital has more than 400 beds with different units and clinics, which include medical wards, pediatric ward, psychiatry ward, surgery, emergency ward, chemotherapy center, gynecology and obstetrics ward, and HIV clinic. In addition, the hospital provides outpatient medical services to 250,000 patients. Besides, the hospital serves as a teaching hospital, research center, and referral center for more than five million people.

### 2.2. Study Design

The study was conducted using a prospective hospital-based observational study.

### 2.3. Population

#### 2.3.1. Study Population

All patients admitted to the medical ward taking antibiotics during the study period and who volunteered to take part in the study were the study population.

### 2.4. Inclusion and Exclusion Criteria

#### 2.4.1. Inclusion Criteria

Patients whose age was above 18 years old who had received an IV antibiotic for more than 48 hours, were able to eat or tolerate enteral feeding, patients with a gastrointestinal tract, absence of bowel abnormalities, and who adequately absorbed oral medications via the oral, gastric, or nasogastric tube route were included in the study.

#### 2.4.2. Exclusion Criteria

For those patients who received IV prophylactic antibiotics, a prolonged course of IV antibiotics was required in the case of osteomyelitis, meningitis, *Staphylococcus aureus* bacteremia, and endocarditis. Patients unable to respond to oral medication, patients with grade three and above mucositis, patients with unstable conditions, patients refusing oral medications, and immunocompromized patients (febrile neutropenia on cancer chemotherapy) were excluded from the study.

### 2.5. Sample Size Determination and Sampling Technique

Because no research had been published on this topic in Ethiopia before October 2021, we took a 50% prevalence of IV to PO conversion. The sample size was calculated based on the single population proportion formula using the following assumption: (1.96) 2 were used *Zα*/2 and the proportion (P) of IV to PO antimicrobial conversion in these groups with a 95% confidence interval (CI) and marginal error (d) of 5%.

(1) $$\begin{array}{c} n=\frac{Z_{\alpha /2}{}^{2}P\left(1-P\right)}{d^{2}}=384,\\ X=\frac{N\times n}{N+n},\\ X=\frac{\left(1530x384\right)}{\left(1530+384\right)}=\frac{587520}{1914}=307+16. \end{array}$$

*X* = 307.5 population plus 5% (m) contingency = 16, *N* = 1530 population, and *n* = 384 population.

**Figure 1:**
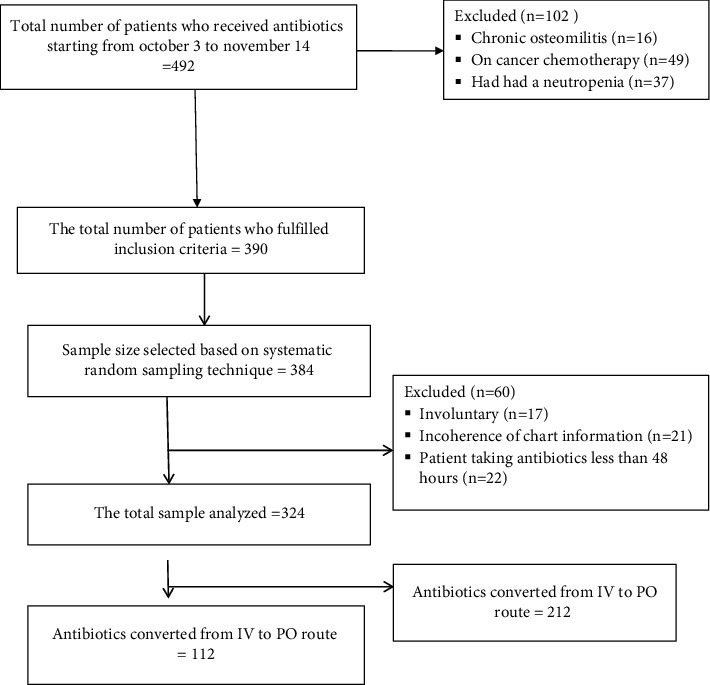
The procedure of sample size calculation on IV to PO conversion practice of antibiotics at the University of Gondar Specialized Hospital from October 3 to November 14, 2021 (*n* = 324).

Where *n* = the number of calculated patients from the aforementioned formula. *N* = the number of patients expected to be admitted within three months in the ward. Based on the above formula, assumptions, correction formula, and 5% of contingency, the sample size (n) was calculated to be 323.5. Finally, we selected 324 patients using a systematic random sampling technique. Then, every third patient who arrived at the clinic was selected for the study. The following figure shows the procedure of how to exclude and include the study participant during the study period (Figure 1).

### 2.6. Study Variable

#### 2.6.1. Dependent Variable

IV to PO conversion practices.

#### 2.6.2. Independent Variables

- Demographic data: sex, age, allergy, presumed or documented diagnosis, culture, and comorbidity
- Types of antibiotics: ceftriaxone, metronidazole cloxacillin, ceftazidime ciprofloxacin, and vancomycin
- Duration of antibiotics administered
- Length of hospital stays
- Clinical parameters: swallowing status, tolerance of oral fluid, and clinical improvement

### 2.7. Data Collection Technique and Instrument

#### 2.7.1. Data Collection Procedure

Data were collected using a pretested structured questionnaire by recruiting data collectors. The data abstraction format had three parts. The first part contains demographic characteristics of patients, comorbidities, allergies, primary diagnosis or presumed indication for antibiotic therapy, and microbiological results. The second part contains the type of antibiotics administered, route of administration, duration of IV therapy, and length of hospital stay. The third part is collected from the patient's chart and files and contains the daily recording of signs and symptoms to assess clinical stability throughout the hospital stay.

#### 2.7.2. Data Quality Assurance

To assure the quality of the data, a pretest was done on 10% of the sample (32 patients) in the data abstraction format before the main data collection was done. The pretested data were not included in the study, and the appropriate adjustment was done to the data abstraction format. In addition to this, the authors supervised the data collector during the data collection process. The collected data were checked for completeness and consistency on a daily basis.

*(1).* Ethical Approval and Consent to Participate. Ethical clearance with ethical approval code SOPs/117/2021 was obtained from the ethical clearance committee of the department of clinical pharmacy, school of pharmacy, University of Gondar. Permission to access the medical records of patients was then obtained from the UOGCSH clinical directorate. This study was conducted following the Declaration of Helsinki, in which it is stated that in medical research using identifiable human material or data, physicians must normally seek consent for the collection, analysis, storage, and/or reuse. Confidentiality of the information regarding patients was ensured in such a way that the data was only used for the study purpose. To ensure privacy and confidentiality, information collected was not directly linked to the respective participants; names of patients were not used. Codes were used as an identifier.

#### 2.7.3. Data Processing and Analysis

The data was entered into a computer database using Epi-data version 4.6 and exported to STATA version 14.2 for analysis. Categorical variables were summarised as frequencies and proportions. Continuous variables were first to be tested for normal distribution using a histogram. All continuous variables were normally distributed. Simple descriptive statistics such as mean ± SD, frequency, and percentages were determined. A bivariable logistic regression analysis was done to see the association between the independent variable and the outcome variable. Then, independent variables having a *P* value of less than 0.2 were included in multivariate logistic regression analysis to identify independent predictors of IV to PO conversion. Those variables having a *P* value of <0.05 were considered statistically significant. The Hosmer–Lemeshow goodness of fit test for logistic regression was done, and the model was well fitted for IV to PO conversion (*P*=0.95). A contingency coefficient test was done to see whether categorical variables have multicollinearity. A variance inflection factor was used to test whether continuous variables had multicollinearity and had no correlation between independent variables.

### 2.8. Operational Definitions

There are mainly three types of IV to PO conversions [8].

- Sequential therapy: the act of replacing a parenteral version of medication with its oral counterpart of the same compound.
- Switch therapy: the conversion of an IV medication to a PO equivalent that is in the same class and has the same level of potency but is a different compound.
- Stepdown therapy: the conversion of injectable medications to oral medications in another class or to different medications within the same class where frequency, dose, and spectrum of activity may not be the same. It converts IV to PO agents with reduced potency.

## 3. Results

### 3.1. Sociodemographic and Clinical Data of the Patients

Out of 324 study participants, 63.3% were males. The majority of them were in the age group of 18–29 (35%). The most frequent site of infection was soft tissue infection (SSI) 106 (32.7%), followed by respiratory tract infection 101 (31.2%). Among enrolled patients, 127 (39.2%) had one or more comorbidities. The major comorbidities encountered were tuberculosis (TB) 18(12.3%), hypertension 16 (10.9%), and other 47 (32.2%). The mean duration of antibiotics was 8.6 days and the mean length of hospital stay (LOHS) was 10.8 days (Table 1).

### 3.2. Types of Prescribed Medications and Antibiotic Conversion Practice

**Figure 2:**
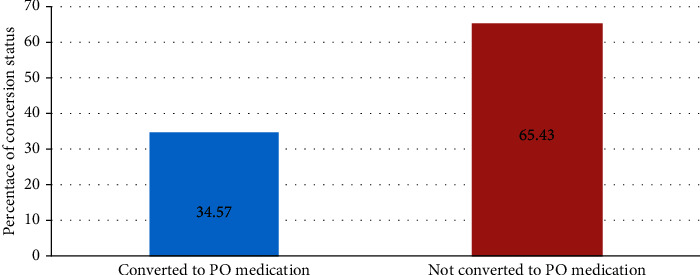
Intravenous to per oral conversation practices at the University of Gondar Comprehensive and Specialized Hospital from October 3 to November 14, 2021 (*n* = 324).

**Figure 3:**
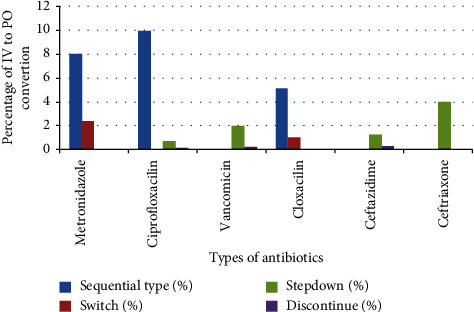
Types of IV to PO conversion practice at the University of Gondar Compressive Specialized Hospital from October 3 to November 14, 2021 (*n* = 324).

**Figure 4:**
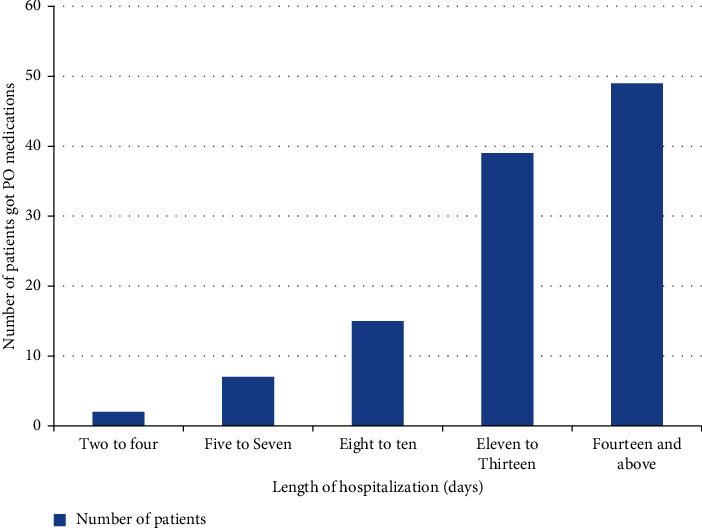
Percentage of IV to PO conversion based on length of hospital stay at the University of Gondar Compressive Specialized Hospital from October 3 to November 14, 2021 (*n* = 324).

The most frequently prescribed antibiotics used for empiric treatment were ceftriaxone 226 (45.4%), followed by metronidazole165 (33.2%), and cloxacillin 57 (11.4%) (Table 2). A total of 324 patients were on IV antibiotics courses, and they met the eligibility criteria and were evaluated. Of the 324 antibiotics courses, 212 (65.43%) were not converted to the PO route and IV antibiotics, and 112 (34.57%) were converted to the PO route of administration and met the criteria for conversion (Figure 2). The most frequent type of conversion practice was the sequential conversion of 75 (23.1%), followed by a switch of 24 (7.4%) (Figure 3). Based on the length of hospital stay, antibiotics are mostly not converted peroral early. As hospital stays increased, the practice of IV antibiotics to PO conversion was increased. After two weeks, IV to PO conversion practice has greatly increased (Figure 4).

### 3.3. Determinates Intravenous Antibiotic to Peroral Antibiotics Conversion Practice

In bivariate logistic regression analysis, 9 variables had a *P* value less than 0.2 and were candidates for multivariate logistic regression analysis. Tachypnea, unavailability of the medication, longer length of hospital stays, presence of comorbidity, and higher temperature significantly affected IV to PO conversion in multivariate logistic regression. Accordingly, the presence of tachypnea increased the risk of IV medication not being converted to PO medications by an odds of 1.89 as compared to patients without tachypnea (AOR1.89, 95% CI1.9–5, *P*=0.0001). The presence of higher body temperature increased the risk of IV medication not being converted to PO medications by an odds of 1.43 as compared to afebrile patients (AOR 1.43, 95% CI1.33–3.23, *P*=0.034). The unavailability of medication increased the risk of IV medication not being converted to PO medications by an odds of 3.27 as compared to the availability of the medication (AOR 3.27, 95% CI1.42–7.53, *P*=0.005). Patients with a hospital stay greater than 10.78 days increased the risk of IV medication not being converted to PO medications by an odds of 1.82 as compared to patients with a hospital stay ≤10.78days (AOR 1.82, 95% CI1.7–4.7, *P*=0.039). The presence of comorbidity increased the risk of IV medication not being converted to PO medications by odds of 2.74 as compared to patients without comorbidity (AOR 2.74, 95% CI1.9–7.6, *P*=0.026) (Table 3).

## 4. Discussion

This study aimed to evaluate the practice of conversion from IV to PO antibiotic conversion and its associated factors. The prevalence of IV to PO conversion practice in this study was 34.5%, of which 23% was the sequential type (23%), followed by switch types of conversion (7.4%).

The prevalence of IV to PO antibiotic conversion practice in this study was higher than in the study conducted in India. This difference could be due to the fact that the India study included a sampler sample. In addition to the above factors, the availability of oral formulation antibiotics, the knowledge and practice of prescribers, and healthcare policy are the determinant factors of conversion practice of IV to oral PO of administration. On the other hand, a lower practice of IV to PO conversion practice was reported in Lebanon at 26.1% [19]. This difference might be due to the nature of the methods employed in the Lebanese study, which was a retrospective study that might have led to the missing of important information during data collection [20].

Although patients were eligible for conversion of antibiotics from IV to PO formulations, 63.43% of antibiotics were not converted to oral formulations. This implies that the practice of IV to PO conversion practice in the University of Gondar Comprehensive Specialized Hospital was poor. In this regard, patients were exposed to several complications *w*, including spasms, pain, hematoma, infiltration, air embolism, overhydration, and an increased risk of cannula-associated and catheter-related adverse events [21].

For those patients who were not converted to PO medication, the cost of the IV route is comparably higher than the cost of PO counterparts. The cost of IV medication was increased unless the antibiotics were converted to PO medications [7, 22]. This result was supported by a Michigan study that reported that patients who had converted their antibiotics from IV to PO were saving $15,000 in drug costs [23]. In addition, early conversion from IV to PO can reduce hidden costs. References [7, 22].

In this study, the most frequent type of IV to PO conversion practice was sequential type 75 (23%) followed by the switch type of conversion 24 (7.4%). This study was different from a study conducted in India that reported the most frequent type of conversion practice was a step-down (45.3%) and a sequential type (18.7%) [24]. This difference might be due to most drugs not being available by oral formulation of the same type at the University of Gondar Compressive Specialized Hospital. In this study, cloxacillin was one of the frequently prescribed medications (17.6%). This could be due to most of the admitted patients being diagnosed with skin and soft tissue infections (32.7%). This finding was supported by a study conducted in Michigan which reported that cloxacillin and penicillin derivatives were the predominant drugs available in resource-limited country hospitals [23]. Regarding cloxacillin, the availability of PO and IV formulations was adequate in the study area; healthcare professionals were converted sequentially. This might be the reason for the sequential type of conversion being the most frequent type of conversion practiced in this study.

In this study, the mean length of hospital stays was 10.78 ± 6.6 days. This was a longer hospital stay as compared to a study conducted in Lebanon which reported a shorter mean length of hospital stay (6.67 ± 2.74) days [19]. This difference might be due to the IV to PO conversion practice of antibiotics in this study being lower. In this regard, the study participants incurred additional costs due to increased hospital stays [24].

Regarding the associated factor of IV to PO conversion practice, the presence of tachypnea is significantly associated with IV medication not being converted to PO medication. This study was in line with a study conducted in the Netherlands [25]. This might be due to this factor's being a significant clinical parameter for the conversion of IV to PO medication.

The unavailability of suitable oral medication was significantly associated with IV medication not being converted to PO medications as compared to the availability of the medication. This may be due to the fact that the number of administered antibiotic courses of treatment that were switched to a suitable oral dosage form was very small and the involved antibiotics were mostly macrolides, fluoroquinolones, metronidazole, ceftriaxone, vancomycin, and ceftazidime classes. These antibiotics were converted in a sequential type to oral formulations [26].

Higher temperatures are significantly associated with IV medication not being converted to PO medications compared to patients with normal temperatures. This factor was in line with a study conducted in the Netherlands [25]. Patients with a hospital stay greater than 10.78 days were significantly associated with IV medication not being converted to PO medications as compared to patients with a hospital stay ≤10.78 days. This factor was in line with a study conducted in Asia [26]and India [27]. Conversion of IV to PO therapy can reduce the length of hospital stay, healthcare costs, and risk of complications related to IV therapy [27]. The presence of comorbidity is significantly associated with IV medication not being converted to PO medications as compared to patients without comorbidity. This factor was in line with a previous study conducted in China [28] and Jimma University Hospital [29].

### 4.1. Strength and Limitation

#### 4.1.1. Strength of the Study

The prospective nature of the method increases the quality of data as it enables us to follow the patients till discharged.

#### 4.1.2. Limitation

It is a single-center study and cannot be generalized to other hospitals. In addition, the cost of medications was not assessed as it may have affected conversion practice.

## 5. Conclusion

The findings of this study showed that IV to PO conversion practice was frequent. The most frequent conversion practice was sequentially followed by switch conversion. Tachypnea, unavailability of medication, higher temperature, hospital stay of more than than 10.78-days, and comorbidity were all significantly associated with IV to PO conversion practice. Awareness of IV to PO conversion practice and short-term training of healthcare teams is vital for better antibiotic conversion practice.

### 5.1. Recommendation

Based on the findings of our study we forwarded the following recommendations:

#### 5.1.1. To University of Gondar Compressive Specialized Hospital

Create awareness about IV to PO conversion practice by preparing for hospital-specific IV to PO conversion.

#### 5.1.2. To Ministry of Health

To PO conversion practice guidelines to be included in the curriculum and short-term training and create awareness for healthcare teams at the national level.

## Figures and Tables

**Table 1:** Sociodemographic and clinical data of study participants at the University of Gondar Specialized Hospital from October 3 to November 14, 2021 (*n* = 324).

Variable	Category	Frequency	Percent (%)
Sex	Male	205	63.3%
	Female	119	36.7%

Age	18–29	112	34.6%
	30–41	84	25.95%
	42–53	45	13.9%
	54–65	36	11.1%
	≥66	47	14.5%
	Mean age	41.4 ± 18.6 (SD)	—

Allergy	Yes	3	0.9%
	No	321	99.1%

Culture	Yes	12	3.7%
	No	322	96.3%

Comorbidity	Yes	127	39.2%
	No	197	60.8%

Types of comorbidity	DM	10	6.84%
	HTN	16	10.95%
	CHF	16	10.95%
	RVI	16	10.95%
	Stroke	15	10.27%
	TB	18	12.33%
	Fracture	8	5.48%
	Others	47	32.22%

Diagnosis	RTI	101	31.2%
	COPD exacerbation	22	6.8%
	UTI	61	18.8%
	SSI	106	32.7%
	GTI	14	4.3%
	CNS infection	6	1.9%
	Other infection	14	4.3%

Duration of antibiotics	8.6 ± 4.98 (SD)	—	—

LOHS	10.78 ± 6.6 (SD)	—	—

**Table 2:** SEQ Table \∗ ARABIC Types of prescribed medications at the University of Gondar Comprehensive and Specialized Hospital from October 3 to November 14, 2021 (*n *=* *324).

Medications	Frequency	Percent (%)
Ceftriaxone	226	45.4
Metronidazole	165	33.2
Cloxacillin	57	11.4
Vancomycin	26	5.2
Ceftazidime	16	3.2
Ciprofloxacin	8	1.6

**Table 3:** Logistic regression analysis of antibiotics conversion practice at UOGCSH (*n* = 324).

Variable	Category	Not converted to PO		COR (95%CI)	*P* value	AOR (95%CI)	*P* value
		Yes	No				
Physician experience	Specialist	5	11	1		1	
	Intern	121	62	4.3 (1.43 –12.9)	0.009	3.91 (.92–16)	0.064
	GP	58	41	3.11 (1.005–9.64)	0.049	1.82 (0.41–8.1)	0.43
	Resident	9	17	1.16 (0.3–4.4)	0.82	0.31 (0.046–2.1)	0.23
Febrile	No	46	68	1		1	
	Yes	147	63	3.45 (2.14–5.55)	0.0001	1.43 (1.33–3.23)	0.034^*∗*^
Tachypenia	No	67	98	1		1	
	Yes	126	33	5.58 (3.4–9.14)	0.03	1.89 (1.9–5)	0.0001^*∗*^
Hypotention	No	54	78	1		1	
	Yes	139	53	3.8 (2.4–6)	0.0001	2.55 (0.13–5.7)	0.42
Tachycardia	No	58	56	1		1	
	Yes	135	75	1.73 (1.09–2.76)	0.019	1.7 (0.76–3.7)	0.189
Availability of medication	Available	139	80	1		1	
	Not available	54	51	1.64 (1.024–2.63)	0.039	3.27 (1.42–7.53)	0.005^*∗*^
Leucocytosis	No	107	113	1		1	
	Yes	86	18	5.045 (2.84–8.94)	0.006	3.36 (0.34–8.4)	0.53
Length of hospital stay (mean ± SD)	≤10.78 ± 6.6	73	115	1		1	
	>10.78 ± 6.6	120	16	11.8 (6.5–21.5)	0.01	1.82 (1.7–4.7)	0.039^*∗*^
Comorbidity	No	107	113	1		1	
	Yes	94	10	9.93 (10.5–14.8)	0.0002	2.74 (1.9–7.6)	0.026^*∗*^

^∗^Significance (*P* < 0.05). Abbreviation: SD: standard deviation; GP: general practitioner.

## Data Availability

The datasets used and analyzed during the current study are available from the corresponding author upon reasonable request.
